# Failed Switching off in the MIBI-Parathyroid Scintigraphy in a Dialyzed Patient with Secondary Hyperparathyroidism Responsive to Cinacalcet Therapy

**DOI:** 10.1155/2010/206801

**Published:** 2010-06-27

**Authors:** Piergiorgio Bolasco, Alessandra Serra, Maurizio Loi, Andrea Galfré, Mario Piga

**Affiliations:** ^1^Territorial Nephrology and Dialysis Department, ASL, 8 Cagliari, via Turati 4/C-Quartu Sant'Elena, Cagliari, Sardinia, Italy; ^2^Nuclear Medicine Department, Azienda Ospedaliero-Universitaria di Cagliari, 09124 Cagliari, Italy; ^3^Radiology Department, Azienda Ospedaliero-Universitaria di Cagliari, 09124 Cagliari, Italy

## Abstract

The aims of your case report is to show the predictivity of ^99m^Tc-sestamibi (MIBI) scintigraphy and doppler ultrasound imaging on secondary hyperparathyroidism (SHPT) in a patient responsive to calcimimetic treatment. Moreover, it has been reported that calcimimetic has great potential in reducing the volume of the parathyroid gland. On the other hand, the MIBI scintigraphy is considered a crucial diagnostic procedure to monitor the response to therapy in terms of turnover and cellular metabolism; whereas, ultrasound to monitor the volume variation in response to treatment. It is described the case of a 73-year-old man on hemodialysis from 1995 for ESRD. Within 2 years the patient gradually developed SHPT with progressively increased iPTH up to 1,000 *ρ*g/ml. The ultrasound, highlighted the presence of two parathyroid hyperplasia, confirmed by scintigraphy, showing focal increase uptake of sestamibi in the same anatomical areas. As a result of the patient's refusal to perform a parathyroidectomy, cinacalcet, was administered (65 mg overage daily dose). After a year of treatment, there was a striking decrease of iPTH (from 1300 to 57 *ρ*g/ml, −95%); but, on the contrary to expectations, this positive metabolic outcome, was not followed by parathyroid changes in ultrasound and scintigraphic findings.

## 1. Introduction

Over the past two years, the introduction of new drug molecules, including new phosphate binders, less-calcemic vitamin D analogues and calcimimetic, have been undergoing an incisive therapeutic change in the treatment of secondary hyperparathyroidism in haemodialysis patients. The novel vitamin D analogs, paracalcitol, doxercalciferol and others as well as calcitriol and alfacalcidol, all decrease serum PTH efficiently, but they also have a tendency to increase serum phosphorus and serum calcium [1]. Cinacalcet is a type II calcimimetic agent, that acts as an allosteric modulator of the calcium-sensing receptor, which is present on the surface of parathyroid cells, thus providing a new means of regulating PTH secretion, by amplifying the receptor's sensitivity to extracellular calcium and reducing PTH concentrations. Cinacalcet has the same marked decrease in serum PTH as active vitamin D sterols; however, it causes a decrease of serum calcium and a minor reduction in serum phosphorus [2]. Sesta MIBI parathyroid scintigraphy is a routine imaging technique in the assessment of haemodialysis patients, not only to identify the autonomous glands requiring parathyroidectomy, but also to detect those hyperplastic parathyroids which can be suppressed by medical treatment. 

A clinical case of full biochemical response to calcimimetics therapy with successive regression in parathyroid volume and functionality not being present was reported.

## 2. Case History

A 73-year-old man, began dialysis treatment in 1995, as a result of a 10-year record of chronic kidney disease (CKD) stage II, caused by hypertensive nephroangiosclerosis. The technique of extracorporeal therapy consisted of an endogenous hemodiafiltration (HFR) with polyethersulfone membranes, rythm dialysis was four hours x 3/weeks dialysate: Na: 140 mmol/L, K: 2 mmol/L, bicarbonate: 30 mmol/L, and calcium: 1.5 mmol/L.

In 1997 a mild secondary hyperparathyroidism (SHPT) was detected and serum intact parathyroid hormone (iPTH) was repeatedly found at 386.5 *ρ*g/ml, (normal range 10 to 65 *ρ*g/ml, IRMA Scantibody Radim) with no associated specific symptoms. Checks by Chest X-ray, echocardiogram high-resolution computerized tomography (CT) reported an early detection of large and small vessels calcifications, including sclerodegenerative phenomena on aortic and mitral endothelium valves, despite patient dietary cooperation (there was rare evidence of hyperphosphoremia in over 10 years of dialysis also owing to a large use of sevelamer and calcium carbonate). The patient presented other comorbidities: osteoporosis and severe arthritis (former semiprofessional athlete); an early dialysis amyloidosis with consequent Baker's cyst located in the popliteal fossa, positive for amyloid. In the same period and as a consequence of the same cause, the patient underwent bilateral neurolysis of the median nerve for carpal tunnel syndrome. No liver disease or haematologic and autoimmune diseases were present. 

**Figure 1:**
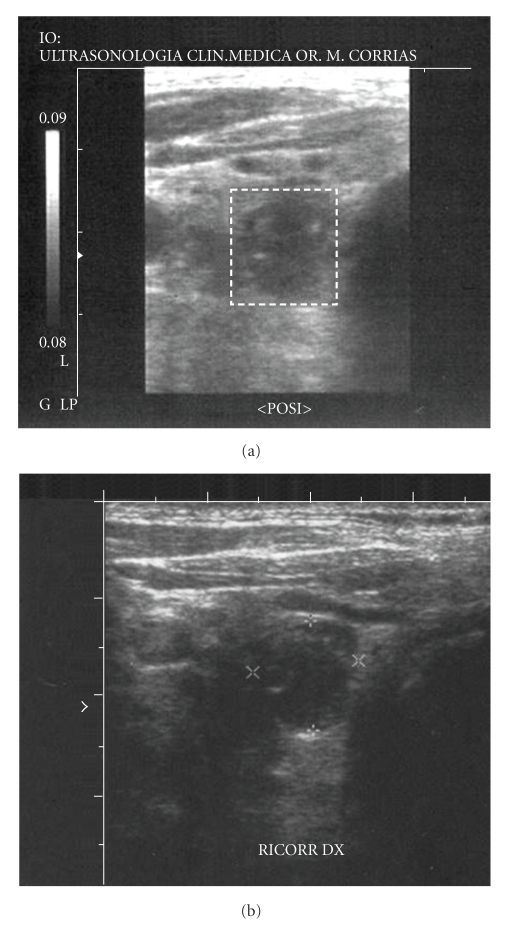


**Figure 2:**
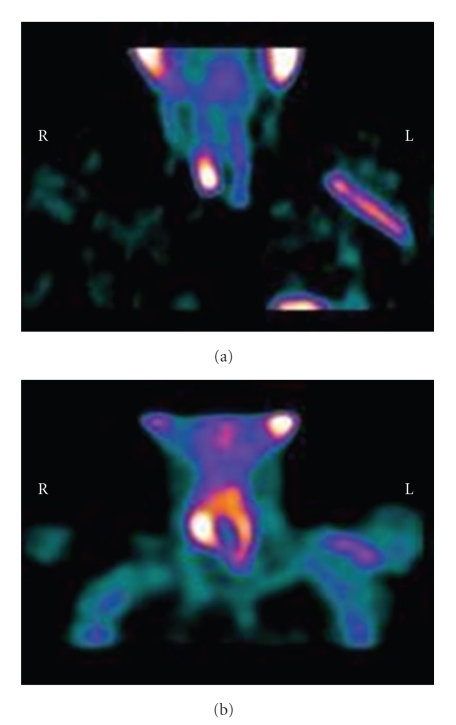


From 2001 to 2004 the patient started oral pulses therapy with calcitriol at dose of 1 *μ*g three times a week without any change in iPTH levels (iPTH = 500 *ρ*g/ml). Due to a lack of response to treatment, calcitriol therapy was given intravenously at the same dose (1 *μ*g three times/week). The iPTH showing a hyperfunctioning parathyroid gland and continued to rise and after a year it rose up to values of 1,000 *ρ*g/ml and serum alkaline phosphatase activity also increased progressively (see Table 1). Doppler ultrasonography showed two enlarged inferior parathyroid glands with hypervascularity (Figure 1), and two hot spots were detected at the same location by ^99m^Tc-MIBI scintigraphy (Figure 2). Surgical treatment (subtotal parathyroidectomy) was proposed, but the patient repeatedly refused; therefore, we decided to initiate therapy with cinacalcet. Before beginning cinacalcet, and without changing conventional therapy (a constant dose of vitamin D and phosphate binders and receiving a stable dialysate calcium concentration), blood tests were performed to determine complete blood counts, protein electrophoresis, 25(OH)_2_D_3_, iPTH, calcium, phosphorus, ionised calcium concentration, and bone turnover markers (bone-specific alkaline phosphatase, osteocalcin). Parathyroid MIBI scintigraphy and ultrasound scanning were done at baseline, and after a year of treatment to evaluate glandular function and structure (Tables 2(a) and 2(b)). In November 2005 oral treatment with cinacalcet was initiated at a daily dose of 60 mg.

The patient continued to receive the same low dose of vitamin D sterols and phosphate binders. 

During the 12 months, conventional therapy (phosphate binders, vitamin D sterol and calcium supplements) and cinacalcet was adjusted, in order to try to achieve and maintain National Kidney Foundation Kidney Disease Outcomes Quality Initiative (NKF-K/DOQI) (targets for PTH (150–300 *ρ*g/ml), serum calcium (8.4–9.5 mg/dl), phosphorus (3.5–5.5 mg/dl), and calcium-phosphorus product (<55 mg^2^/dl^2^)). Dialysate calcium concentration and the size of the dialyzer membrane surface area were not to be changed. In a year of closed followup, serum iPTH values progressively decreased with a good control of serum phosphorus and normal serum calcium values persisted. In December 2006, after a year of treatment with cinacalcet, iPTH levels were found at 57 *ρ*g/ml with a reduction of −95% from the starting value (see Table 1).

Following neck ultrasound performance, after 12 months of cinacalcet therapy, there was the confirmation of the presence of two inferior parathyroid enlargements. The size and echographic features of these lesions remained unchanged, compared with the ultrasound images obtained before treatment. Similarly, ^99m^Tc-MIBI parathyroid scintigraphy confirmed hyperfunctioning parathyroid tissue (Table 2(b)). Further follow-up was not made possible due to the patient's death of myocardial infarction three months later.

## 3. Discussion

Clinical evaluation of SHPT is usually carried out by the assessment of biochemical parameters and morphological and functional imaging. In recent years, various techniques including ultrasound (US), CT, magnetic resonance and MIBI-scintigraphy have been used to localize better enlarged parathyroid glands in patients with SHPT, although MIBI scintigraphy appears to be the most specific in recurrent/persistent SHPT [3–5]. Calcimimetics have become available for the treatment of dialysis patients with insufficiently controlled SHTP as well as calcium and/or phosphate levels on conventional therapies. Cinacalcet belongs to calcimimetic type II compounds that can interact with the calcium-sensing receptor CaSR, increasing its affinity to calcium. Clinical studies have proved cinacalcet to be effective in reducing calcium, phosphate, and PTH levels in haemodialysis patients with severe SHPT [6–9]. Colloton et al. [10] reported that cinacalcet inhibits the progression of parathyroid cell proliferation in rats. Meola et al. [11] described morphological ultrasound variations in hyperplastic parathyroid glands in haemodialysis patients treated with cinacalcet. They observed changes in volume, vascularisation, and sonographic patterns after 12–18 months of therapy and volume reduction was more evident in gland with basal volume <0.5 cm^3^. In agreement with these findings, parathyroid size regression after cinacalcet treatment has been recently reported in [12]. The literature is not yet clear if the inhibitory effect of cinacalcet on PTH secretion is associated with an effective control of parathyroid cell metabolism turnover, specially in parathyroid >0.5 cm^3^ [13]. In our knowledge no data has been reported about the relationship between metabolic changes and MIBI uptake in haemodialysis patients treated with calcimimetic therapy; MIBI is a lipophilic radiotracer that becomes concentrated in cells and in mitochondria through active transport and passive diffusion. The exact mechanism of its elective uptake in abnormal parathyroid glands remains debatable. The size of abnormal parathyroid does not represent the only determinant of MIBI uptake but other factors including the mitochondria numbers, degree of cellular activity, and glycoprotein P expression have been described in [14–17]. Piga et al. [18] stated that MIBI scans do not simply reveal parathyroid enlargement but rather identify hyperfunctioning parathyroid glands in patients with SHPT. Additional evidence that MIBI shows the functional status of parathyroids is found in the correlation between MIBI uptake and intact PTH in SHPT [19, 20]. Moreover, Torregrosa et al. [21] demonstrated that high MIBI uptake is correlated with the G_2_/S (active growth) phase.

It was shown that MIBI-scan could be used to evaluate parathyroid function as well as response to treatment with calcitriol [22, 23]. In our case with positive basal MIBI-scan after the inhibition test with calcitriol (2 *μ*g i.v. three times/week for two weeks after haemodialysis), we could suppose two different scintigraphic findings: (1) lack of parathyroid MIBI-uptake suggestive of a reduction in metabolic activity of parathyroid glands, selecting the responders, and (2) persistence of MIBI uptake indicative of parathyroid functional autonomy, selecting the candidate patients for surgery. 

In our patient the calcitriol, administered in low weekly not pulsing dose to correct hypocalcemia induced by calcimimetic, was not able to inhibit parathyroid hyperplasia. There were not detected high levels of phosphorus as a factor in stimulating hyperplasia [24]. In our case is excluded another but uncommon possibility that ultrasound and scintigraphic could be explained due to a mutation in enzyme CYP3A4 or described using other inhibitor drugs (e.g., ketoconazole, erythromycin, itraconazole) on this enzyme which could interfere in the therapeutic efficacy of calcimimetic [25]. We conclude that a ^99m^Tc-MIBI parathyroid scan could not have showed a good correlation to the response of the cinacalcet treatment. In our case contrary to allexpectations unchanging parathyroid MIBI uptake appears seemingly to be in contrast to the inhibitory effect of calcimimetic, on parathyroid hormone secretion. Possible hypothesis to explain this paradoxical result may be the effect of cinacalcet on Ca receptors. In fact this drug increasing the membrane sensitivity to cations, as calcium, my induce a consequent increase of other cations, as MIBI, resulting in abnormal parathyroid uptake of MIBI independent of a real cellular function.

In conclusion in our case report we believe that ^99m^Tc-MIBI scintigraphy remains to be defined as a useful method in monitoring and predicting efficacy in long-term cinacalcet therapy, in uremic patients; as well, it remains to be defined the meaning of sestamibi uptake, as an index of metabolic turn-over of the parathyroid glands. Instead doppler ultrasonography may represent a good marker to evaluate the regression of volume glandular. However; up until present day, there is not any clear illustration of a modification of the metabolic turnover of the parathyroid gland produced by using calcimimetic agents: surgical and histopathological confirmations should be necessary in more cases.

## Figures and Tables

**Table 1:** Outcome of haematochemical and therapeutic parameters.

	*Start *December 2005	January–March 2006 (monthly average)	April–June 2006 (monthly average)	July–September 2006 (monthly average)	*End *December 2006
iPTH, *ρ*g/ml	1112	821	156	125	57
alkaline phosp. U/L	173	112	142	108	107
bone Alkal. phosp. U/L	66.4	27.3	15.9	12	10
calcium, mg/dL	9.1	9.3	7.8	8.2	9.4
phosphorus, mg/dL	5.6	4.3	4.9	4.6	4.9
ionized calcium, mmol/L	1.16	1.22	1.15	1.14	1.22
cinacalcet, average g/die	60	60	60	45	30
Calcium carbonate, g/die	2.5	5	5	3.3	1.2
Sevelamer, g/die	11.2	7.7	6.4	6.4	4.8
Calcitriol, *μ*g/sett/os	0.75	0.75	0.75	0.75	0.75

**Table:** (a) Scintigraphic and echographic features at the start before calcimimetic administration

	Basal study
^99m^ *T* *c* *-MIBI scintigraphy:*	*2 parathyroid glands*

scintigraphic features	(1) Focal area of increased radiotracer uptake was present posteriorly to the lower pole of the right thyroid lobe
	(2) Another smaller area of increased radiotracer uptake was present inferiorly to the lower pole of the left thyroid lobe

*CD ultrasound:*	*2 parathyroid glands *

Echographic features	(1) Nonhomogeneous hypoechoic capsulated area with a vascular pole was present posteriorly to the lower pole of the right thyroid lobe. The major three axes of the parathyroid glands were 13.8 × 10.0 × 8.0 mm
	(2) Non homogeneous hypoechoic capsulated area with a vascular pole was present inferiorly to the lower pole of the left thyroid lobe. The major three axes of the parathyroid gland were 8 × 5 × 3 mm

**Table:** (b) Scintigraphic and echographic features after one year of calcimimetic administration

	After 1 year of cinacalcet therapy
^99m^ *T* *c* *-MIBI scintigraphy *	*2 parathyroid glands*

Scintigraphic features	The focal areas of increased radiotracer uptake were unmodified respect to basal control.

*CD ultrasound *	*2 parathyroid glands *

Echographic features	(1) Echographic structure of the lesion present posteriorly to the lower right pole of the thyroid lobe was unmodified. The major three axes of the parathyroid gland were 11 × 10 × 8 mm
	(2) Echographic lesion inferiorly to the lower pole of the left thyroid lobe was unmodified respect to basal control
